# Neomycin Intercalation in Montmorillonite: The Role of Ion Exchange Capacity and Process Conditions

**DOI:** 10.3390/ma17174207

**Published:** 2024-08-25

**Authors:** Alicja Rapacz-Kmita, Marcin Gajek, Magdalena Dudek, Roksana Kurpanik, Stanisława Kluska, Ewa Stodolak-Zych

**Affiliations:** 1 Faculty of Materials Science and Ceramics, AGH University of Krakow, A. Mickiewicza 30, 30-059 Krakow, Poland; mgajek@agh.edu.pl (M.G.); kurpanik@agh.edu.pl (R.K.); kluska@agh.edu.pl (S.K.); 2 Faculty of Energy and Fuels, AGH University of Krakow, A. Mickiewicza 30, 30-059 Krakow, Poland; potoczek@agh.edu.pl

**Keywords:** montmorillonite, intercalation, neomycin, thermal properties, antibacterial activity

## Abstract

The study examined the possibility of intercalation of montmorillonite with neomycin in an aqueous drug solution and the factors influencing the effectiveness of this process, such as the ion exchange capacity and process conditions, including the time and temperature of incubation with the drug. X-ray diffractometry (XRD), infrared spectroscopy (FTIR), thermal analysis (DSC/TG), and Zeta potential measurement were used to confirm drug intercalation as well as to investigate the nature of clay–drug interactions. The obtained conjugates with the most favorable physicochemical properties were also tested for antibacterial response against Gram-negative bacteria (*Escherichia coli*) to confirm that the bactericidal properties of neomycin were retained after intercalation and UV–VIS spectrophotometry was used to examine the kinetics of drug release from the carrier. The results of the conducted research clearly indicate the successful intercalation of neomycin in montmorillonite and indicate the influence of process parameters on the properties of not only the conjugates themselves but also the properties of the intercalated drug, particularly its bactericidal activity. Ultimately, a temperature of 50 °C was found to be optimal for effective drug intercalation and the conjugates obtained within 2 h showed the highest antibacterial activity, indicating the highest potential of the thus-obtained montmorillonite conjugates as neomycin carriers.

## 1. Introduction

Clay minerals, of natural or synthetic origin, are a large group of raw materials, characterized by easy availability, relatively low cost, and unique properties, such as sorption capacity, ion exchange capacity, or the ability to absorb water and ions from aqueous solutions. At the same time, they are stable and inert materials, which allows them to be used in medicine and pharmacy, guaranteeing high biocompatibility [1,2]. Due to their developed internal specific surface, favorable rheological properties, chemical stability, and lack of toxicity, clay minerals such as kaolinite, montmorillonite, saponite, hectorite, palygorskite, and sepiolite are widely used as pharmaceutical raw materials, being part of the assumptions of controlled drug delivery systems [1,3,4]. MMT is a type of clay mineral with a layered sheet-like structure. These layers are only about 1 nanometer thick and they are stacked on top of each other with weak van der Waals forces holding them together. The individual layers themselves are essentially two-dimensional while MMT naturally exists in a 3D stacked form [3].

Drug delivery systems using clay minerals are based on the production of carrier-drug conjugates by intercalation of drug substance molecules in the interlayer spaces, (by penetration of the interlayer spaces of clay minerals by organic molecules) [5,6]. The potential of clay minerals in this area results primarily from the ability to swell and ion exchange, which allow the introduction of the drug into the interlayer spaces, as well as from the fact that numerous interactions occur between the carrier and the drug, which has a beneficial effect on the release profile of the intercalated drug [5]. Due to the multiple interactions of clay minerals with organic compounds and the impact of the carrier on the release kinetics, the bioavailability of the drug over time may vary [2,5]. After intercalation with a drug, montmorillonite can be described as a hybrid 2D/3D nanomaterial. The base structure remains fundamentally 2D but the introduction of intercalated molecules gives it properties that extend into the third dimension. This hybrid nature is crucial for applications such as controlled drug release, where the layered structure helps modulate the release profile [5]. Therefore, it is necessary to conduct research on the use of individual clay carriers for each drug separately.

Among the numerous mechanisms studied for the development of materials with controlled release of drug substances, the phenomenon of ion exchange has attracted considerable scientific attention [7]. Naturally occurring cation exchangers are clay minerals, especially montmorillonite and saponite, due to their high ion exchange capacity (CEC). In the context of drug delivery systems, this creates the possibility of producing clay particle–drug conjugates by ion exchange, by mixing the raw material in the drug solution. In the internal environment of the human body, ion exchange takes place again but in the reverse direction, during which the drug in the form of a cation is released, its place is taken by cations commonly found in the body, while the drug carrier is removed [5].

However, the chemical nature of the interactions of clay minerals with organic molecules may be different and it depends on the type of mineral and the functional groups present in the intercalated compound [8,9]. The conditions of the intercalation process, in particular the initial concentration of the drug in the solution, the ionic strength of the solution, temperature, pH, time, and mixing speed, are of great importance for the formation of interactions between clay minerals and organic molecules [5,6,10].

Attempts to intercalate non-steroidal anti-inflammatory drugs, ibuprofen, naproxen, and diclofenac into the interspaces of montmorillonite have already been described in the literature [11,12,13]. However, the short biological half-life of ibuprofen, as well as the frequent side effects, motivate the development of a more efficient system for its controlled release. In vitro release studies showed more favorable release kinetics at pH 7.4, corresponding to the conditions in the intestines, than at pH 1.2 (pH of gastric content), which supports the use of montmorillonite in ibuprofen-sustained release systems. Rapid metabolism, resulting in a short half-life, also applies to many other drugs, for example, the opioid painkiller tramadol [14], which should be taken at least five times a day to maintain a therapeutic concentration of the drug in the blood. This is particularly unfavorable since pharmacological tolerance to tramadol quickly increases and addiction may also occur. Physicochemical characterization of carriers and in vitro release studies confirmed the potential of montmorillonite for the controlled release of tramadol, which would, to some extent, eliminate the mentioned side effects.

There are also works on the possibility of using montmorillonite as a carrier of a non-selective drug blocking β-adrenergic receptors, timolol, which was successfully introduced between montmorillonite packets. It was found that drug release does not occur to 100%, as ion exchange reactions are equilibrium processes and interlayer cations are never completely exchanged [15]. The literature also includes studies on the intercalation of other β-blockers, propranolol and carvedilol, which confirm the usefulness of montmorillonite in controlled drug delivery systems [16,17].

The literature also reports the preparation of montmorillonite conjugates with drugs for the controlled release of donepezil [18], venlafaxine [19], or nicotine [20]. The tests of the obtained conjugates included the physicochemical assessment of the carriers and the release kinetics of the intercalated drug, which in each case depended on the conditions of the intercalation process, in particular, pH. According to research results [19], the use of montmorillonite as a carrier for venlafaxine creates an opportunity to reduce the frequency of its administration from four to two times a day.

A group of drugs often used for intercalation are antibiotics [21,22,23,24] and antibacterial studies show that after intercalation they retain their bactericidal properties. Studies on the intercalation of metronidazole [25] showed that the interaction of the drug with montmorillonite does not induce any changes in the chemical nature of the drug. Among the antibiotics intercalated into the interlayer spaces of MMT is also gentamicin [26,27,28,29,30,31], one of the few thermally stable antibiotics, belonging, like neomycin, to the group of aminoglycoside antibiotics.

In the field of systems based on clay minerals, research was conducted not only on the intercalation of drugs, but also vitamins. Studies have shown that both natural [32,33] and synthetic [34] montmorillonite can be a carrier of, for example, vitamin B1. In the case of synthetic montmorillonite, it was observed that the chemical composition of the synthesized mineral directly affects the value of the ion exchange capacity, which in turn affects the amount of the intercalated compound. The literature also includes research on the use of polymer composites with the addition of clay minerals to deliver various drugs, including lidocaine [35], ibuprofen [36,37], dexamethasone [38], as well as research on mucoadhesive nicotine carriers [39,40] where the addition of magnesium aluminum silicate modified the nicotine release profile.

The aim of the work was to undertake research on the use of montmorillonite as a carrier of neomycin, a topic that has not been deeply explored in the literature on the subject. Neomycin is a water-soluble and relatively stable alkaline solution (a range of pH 2.0 to 9.0) antibiotic that belongs to the aminoglycosides group of antibiotics. It functions by inhibiting bacterial protein synthesis, primarily against gram-negative bacteria [41]. When ingested orally, neomycin exhibits limited absorption into the systemic circulation, rendering it especially advantageous for addressing issues within the gastrointestinal (GI) tract [42]. Protecting neomycin by intercalating the antibiotic in MMT will allow it to prolong its release time and maintain its therapeutic concentration over a longer period of time. The goal was achieved by obtaining montmorillonite–neomycin (MMT–NS) conjugates by mixing the mineral raw material with an aqueous solution of the drug in the form of neomycin sulfate. The influence of drug preparation process parameters and the initial amount of drug in the solution (different CEC values of the mineral) on the effectiveness of drug intercalation in montmorillonite and on the initial properties of conjugates in the context of controlled drug release was examined. The effectiveness of intercalation was assessed based on X-ray diffractometry and infrared spectroscopy, as well as thermal analysis. An in vitro drug release study was also performed to evaluate the in vitro drug release and thus the ability of montmorillonite to modify the release profile of neomycin toward sustained release, as well as an antibacterial study to inhibit the growth activity of Gram-negative *Escherichia coli* bacteria.

## 2. Materials and Methods

### 2.1. Characteristics of Raw Materials

Pharmaceutical grade magnesium aluminum silicate (montmorillonite—MMT) with the trade name Veegum^®^F from R.T. Vanderbilt Holding Company, Inc. (Norwalk, CT, USA) was used to prepare the conjugates. To determine the ion exchange capacity of silicate, a spectrophotometric sorption method using copper (II) triethylenetetramine from Avantor Performance Materials Poland S.A. (Kraków, Poland) as a reagent was used (Meier and Kahr, 1999 [43]). The determined CEC (cation exchange capacity) of Veegum^®^F silicate was 77.3 ± 1.1 mmol/100 g.

Neomycin is an antibiotic with a medium spectrum of activity against most Gram-negative and some Gram-positive microorganisms and belongs to a large group of compounds called aminoglycosides, the action of which is irreversibly associated with bacterial ribosomes, structures responsible for protein synthesis, by preventing the growth and multiplication of bacterial cells [43]. Neomycin powder (melting point temperature 187 °C) and tablets with antibiotics were stable for periods of two years (at room temperature). Due to the preparation of conjugates in aqueous suspensions, it was necessary to use a water-soluble drug; therefore, neomycin was used in the form of neomycin sulfate (NS) with the chemical formula C_23_H_46_N_6_O_13_·3H_2_SO_4_ from AMARA Sp. z o. o. (Kraków, Poland).

### 2.2. Preparation of Montmorillonite–Neomycin Conjugates

The amount of an organic compound needed to modify a given mass of a mineral with a known ion exchange capacity was determined based on the ion exchange of layered aluminosilicates known from the literature, according to Equation (1) [44,45], as follows:

X = CEC/100∙m∙M/1000(1)

where

- X—amount of modifying compound [g];
- CEC—ion exchange capacity characteristic of the mineral [mmol/100 g];
- m—assumed amount of the mineral [g];
- M—molar mass of the modifying compound [g/mol].

Thus, based on Equation (1), for the known value of the Veegum^®^F, ion exchange capacity, the theoretical amount of neomycin sulfate that could be intercalated in the interlayer spaces of MMT was calculated.

Using a magnetic stirrer, 2 g of montmorillonite was mixed in 50 mL of distilled water with the addition of 0.705, 1.410, and 1.763 g of neomycin sulfate, which corresponded to the values of 0.5, 1.0, and 1.25 CEC of the mineral, respectively. Drug suspensions were mixed with clay at 30, 50, and 80 °C for 2, 12, and 24 h and reference samples were also obtained under analogous conditions by mixing montmorillonite in distilled water without the addition of a drug. After the specified mixing time, the suspensions were poured into containers and then dried at 60 °C for about 72 h. The dried sediments were ground into powder in an agate mortar and the MMT–NS conjugates obtained in the form of powders were used for further research.

The prepared materials differed in color depending on the duration of the intercalation process, temperature, and the amount of intercalated drug and the clear change in color for the conjugates obtained at 80 °C may indicate the slow decomposition of neomycin at this temperature.

### 2.3. XRD Diffractometry

XRD analysis was performed to assess the effectiveness of the intercalation process using the EMPYREAN X-ray diffractometer from PANalytical Malvern (UK) with a copper lamp. Measurements were made with a step of 0.008° in the range of 3–70° of the 2θ angle value. The most representative range of 3–15° 2θ was selected for further interpretation.

### 2.4. FTIR Infrared Spectroscopy

The degree of drug intercalation in the interlayers of montmorillonite was also assessed based on the presence of characteristic functional groups and vibrations of chemical bonds in IR spectra. The spectra were recorded using a Bruker Vertex 70V spectrometer (Bruker Optik GmbH, Ettlingen, Germany) in the range of 400–4000 cm^−1^. The measurements were performed with a resolution of 4 cm^−1^ and a number of scans of 128 using the transmission technique using pellets prepared from 400 mg of potassium bromide (KBr) and 1 mg of the tested powders.

### 2.5. DSC Differential Scanning Calorimetry and TG Thermogravimetric Analysis

DSC curves were recorded using a STA 449 F3 Jupiter thermal analyzer from Netzsch (Ettlingen, Germany), in an air atmosphere, in the temperature range of 40–510 °C with a temperature increase of 10 °C/min. At the same time, thermogravimetric analysis was performed to provide information on the mass loss of the samples due to water loss and the decomposition of neomycin sulfate. Before measurement, the test samples were weighed on an analytical balance with an accuracy of four decimal places.

### 2.6. Zeta Potential

The magnitude of the electric charge on the surface of the drug carriers was determined by characterizing the Zeta potential in the Zetasizer Nano ZS device from Malvern Instruments (Worcestershire, UK). Aqueous powder suspensions were prepared by ultrasonic homogenization of a 0.05 g sample in 100 mL of distilled water. Each sample was measured five times, each counting from 10 to 100 counts. The Zeta potential measurement of neomycin sulfate itself was performed in ethyl alcohol as a measurement in water was impossible due to the good solubility of the drug.

### 2.7. In Vitro Drug Release Study

Drug release testing was performed in vitro by incubating 10 mg of powder in buffered saline solution (PBS) pH 7.4 at 37 °C for 12 days. For each measurement, 2 mL of solution was taken and supplemented with an equivalent amount of PBS buffer to maintain a constant volume of 100 mL. The amount of released neomycin was determined spectrophotometrically using the oxidation reaction of aminoglycoside antibiotics with KMnO_4_ [46,47]. The collected immersion medium was mixed with a 0.01% KMnO_4_ solution in a 1:1 volume ratio and then heated for 30 min at 60 °C. The absorbance value was measured at a wavelength of 290 nm using a Cecil BioQuest CE 2502 spectrophotometer from Cecil Instruments Limited (Cambridge, UK). The concentration of neomycin was determined based on a calibration curve prepared for concentrations in the range of 0–50 μg/mL.

### 2.8. Antibacterial Tests

For antibacterial tests, three disks with a diameter of 6 mm, a height of 1.5 mm, and a mass of 0.1 g were prepared from the conjugate powders obtained at 50 °C, which was considered optimal for their preparation, and pressed uniaxially under low pressure.

The antibacterial activity of montmorillonite–neomycin conjugates was assessed against Gram-negative bacteria *Escherichia coli* (*E. coli*) (ATCC 25922) using the disk diffusion method. Bacterial culture was conducted on Columbia Blood Agar by Oxoid Thermo Scientific (Hampshire, UK) in Petri dishes at a temperature of 35 ± 2 °C, in an atmosphere of 5% CO_2_ for 24 h. Three to five bacterial colonies were suspended in physiological NaCl solution (0.9%) to obtain a suspension with a density of 0.5 on the McFarland scale (approximately 1.5 × 10^8^ CFU). The suspension prepared in this way was inoculated into the medium (Muller Hinton Agar by Oxoid Thermo Scientific UK) using a sterile swab on 100 mm diameter dishes. After drying, disks of montmorillonite–neomycin conjugates were placed on the cultured substrate, and for comparative purposes, the study was also carried out for a pure montmorillonite disk containing no antibiotic and a disk made of paper soaked in a neomycin sulfate solution. After 24 h of incubation at 35 ± 2 °C in aerobic conditions, the diameters of the bacterial growth inhibition zones were measured with calipers, and the test was repeated three times for each sample.

## 3. Results and Discussion

### 3.1. XRD Analysis

Due to the scattering of X-ray radiation on the crystal structure of layered silicates, it is possible to determine the base distance of the mineral based on the position of the recorded diffraction peaks, based on Bragg’s law.

**Figure 1:**
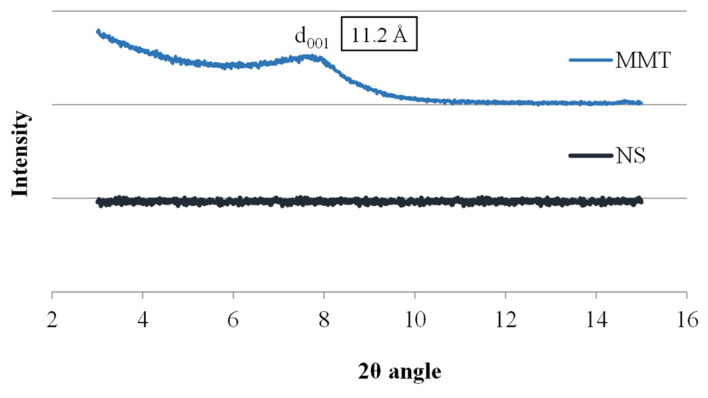
X-ray diffractograms of MMT and NS starting raw materials.

The research began with the analysis of the starting materials used to prepare the conjugates, i.e., montmorillonite and neomycin sulfate alone, and Figure 1 shows X-ray diffractograms for these materials. The diffractogram of neomycin sulfate (NS) showed no peaks over the entire measurement range, which indicates its amorphous nature, while the spectrum of montmorillonite shows a broad peak at 7.75 Å of the 2θ angle, corresponding to the main diffraction peak of MMT, based on which the basal distance of montmorillonite d001 was determined to be 11.2 Å. The thickness of a single TOT (tetrahedral/octahedral/tetrahedral) layer of montmorillonite is 9.6 Å; hence, the interlayer distance of unmodified MMT can be estimated at 1.6 Å.

Preparation of montmorillonite–drug conjugates in an aqueous suspension involved the incorporation of drug molecules between MMT packages, most often accompanied by water molecules. Therefore, the examination of reference samples obtained by mixing montmorillonite with water in conditions analogous to the intercalation process was aimed at checking what effect, especially at elevated temperature, the water present in suspension has on the hydration of montmorillonite and how the accumulation of water in the interlayer spaces associated with hydration increases interlayer distance.

**Figure 2:**
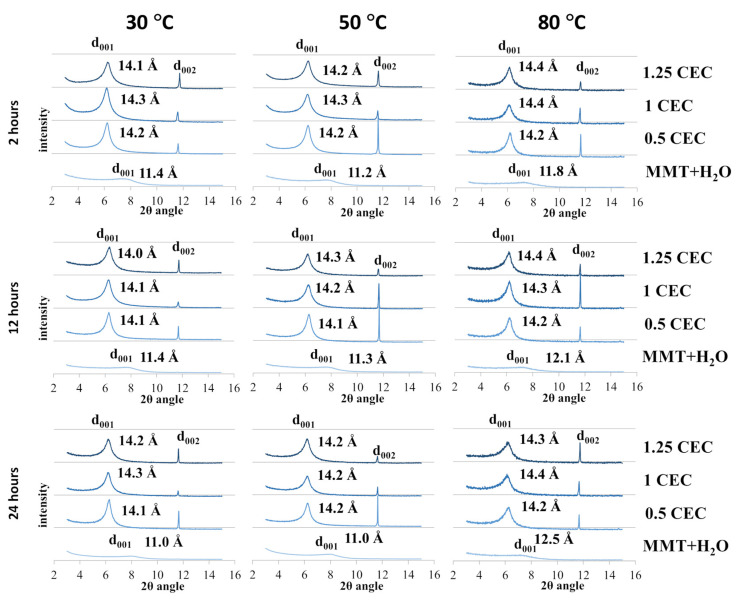
X-ray diffractograms of MMT–NS conjugates and the MMT + H_2_O reference sample obtained under various conditions.

The recorded diffractograms of MMT–NS conjugates and reference samples are summarized in charts for individual conditions of the intercalation and hydration processes and are presented in Figure 2.

In the case of montmorillonite samples mixed with MMT + H_2_O water (reference samples) obtained at 30 °C and 50 °C, regardless of the duration of the hydration process, only a slight increase in the basal distance d_001_ was observed compared to the starting (dry) material. However, a more significant effect of temperature on the degree of hydration of montmorillonite is visible for reference samples obtained at 80 °C, for which the determined basal distances were 0.6–1.3 Å larger compared to the base distance of unmodified montmorillonite. In the case of the highest temperature for obtaining conjugates, there is also a noticeable tendency to increase the d_001_ distance with the mixing time and an increase in the basal distance was recorded to the value of 11.8 Å for 2 h of mixing, 12.1 Å for 12 h, and 12.5 Å for 24 h. The recorded peaks were shifted but their shape and intensity were similar to the diffraction peak of the starting raw material, which may prove that the hydration of montmorillonite increases the interlayer distances but does not cause changes in the crystallinity of montmorillonite.

In the case of the tested samples of MMT–NS conjugates, the diffraction peaks d_001_ diffraction peaks were positioned slightly above 6 Å of the 2θ angle (in the range of 6.12–6.35 Å) and the corresponding basal distances of montmorillonite in the conjugates determined on this basis were approximately 2–3 Å larger than those recorded for the reference samples. The separation of montmorillonite TOT layers manifested by a shift of the characteristic d_001_ peak toward lower values of the 2θ angle, indicating undoubtedly that intercalation of neomycin sulfate into the montmorillonite interlayers occurred.

However, the widening of montmorillonite interlayer spaces occurred to a similar extent for all conjugates, regardless of the conditions in which they were obtained. It is probable then, that neomycin is incorporated between the montmorillonite layers in the form of a monolayer [48] since, taking into account the structure of the neomycin molecule, the differences observed in the basal distances (of the order of 0.1 Å) are too small to indicate a different spatial location of the drug molecules. Convergent observations have been described in publications regarding intercalation between montmorillonite layers of another aminoglycoside antibiotic, gentamicin [26], as well as other drugs [14,21,22,32].

At the same time, it should be emphasized that in individual conjugates, the mentioned monolayer of the drug within the interlayer space may be created by a different number of drug molecules. Therefore, based on the XRD results in the case of neomycin intercalation, the amount of the drug introduced between the montmorillonite layers cannot be assessed.

It should also be noted that the introduction of neomycin sulfate clearly increases the crystallinity of montmorillonite in MMT–NS conjugates. In the case of all conjugates, regardless of the intercalation process parameters, relatively sharp and narrow peaks were recorded compared to the d_001_ peak of unmodified or hydrated montmorillonite. Also, the intensity of the d_001_ peaks of the conjugates was higher than the intensity of the characteristic peak of a neat montmorillonite. This also indicates a change in the size of the crystallites in the MMT–NS conjugates in relation to the crystallites in the starting and hydrated montmorillonite. Similar results have been presented in publications regarding the intercalation of other drugs into the interlayer spaces of montmorillonite [14,15,19,34].

Moreover, in the presence of neomycin, structural changes occur in montmorillonite, manifested by the appearance of an additional d_002_ peak in the diffractograms of the conjugates around 11.6 Å of the 2θ angle, which was absent in the diffractograms of the starting and hydrated montmorillonite. The occurrence of additional peaks has so far been reported in publications describing attempts to intercalate MMT with gentamicin [26].

The recorded X-ray diffractograms of montmorillonite–neomycin conjugates do not differ significantly enough to allow conclusions to be drawn about the optimal conditions of the intercalation process. However, there is undoubtedly a clear shift of the d_001_ peak toward lower values of the 2θ angle compared to the reference samples (MMT + H_2_O), which proves the separation of the montmorillonite layers and confirms the fact that neomycin molecules were incorporated into the interlayer spaces.

### 3.2. FTIR Infrared Spectroscopy

**Figure 3:**
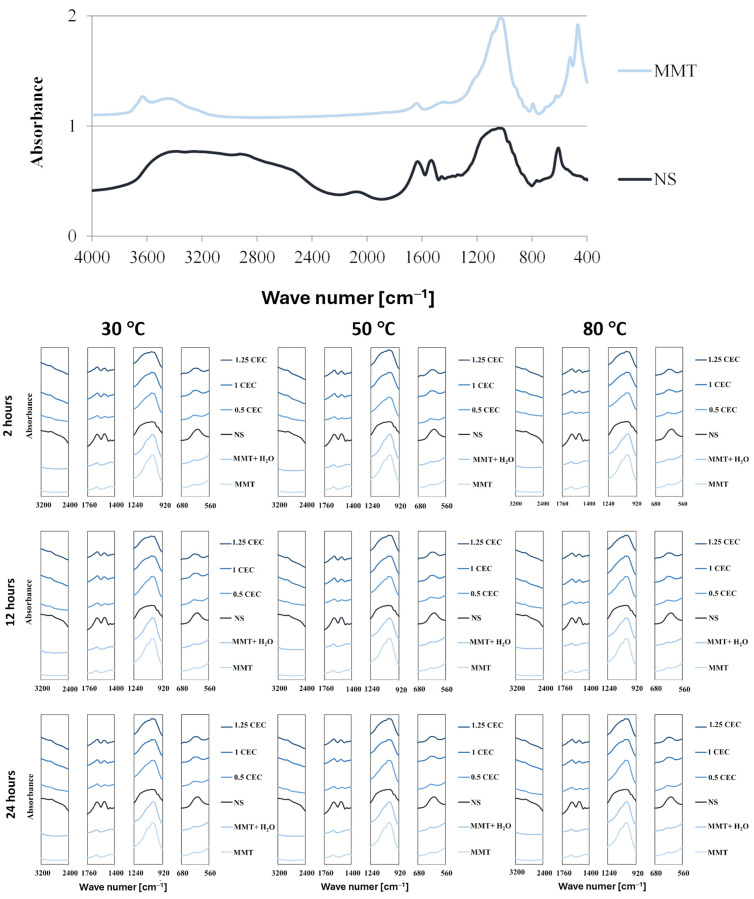
Characteristic sections of FTIR spectra of MMT and NS starting raw materials and MMT–NS conjugates and the MMT + H_2_O reference sample obtained under various conditions.

The presence of the drug in the interlayer spaces of MMT is manifested not only by the enlargement of the interlayer distances of montmorillonite but also by the occurrence of vibrations of chemical bonds typical of organic compounds because of interaction with infrared radiation. Complete FTIR spectra of dry montmorillonite (MMT) raw material before intercalation and neomycin (NS) and characteristic fragments of FTIR spectra of hydrated montmorillonite (MMT + H_2_O) and conjugates (MMT–NS) in the ranges that indicate the presence of the drug in the mineral are shown in Figure 3.

The spectrum of montmorillonite shows bands typical of this mineral, originating from the stretching vibrations of O–H bonds in the Si–OH (3700 cm^−1^) and Al–OH (3620 cm^−1^) groups. Adsorbed water appears in the spectrum at a wave number of approximately 3400 cm^−1^ (O–H stretching vibrations) and approximately 1640 cm^−1^ (H–O–H and O-H bending vibrations). Bands originated from stretching vibrations of Si–O bonds occur around 1035 cm^−1^ and in the range of 450–950 cm^−1^, bending vibrations of Al Al OH (915 cm^−1^) and Al–Mg–OH (850 cm^−1^) groups are visible, as well as deformation of Al–O–Si (520 cm^−1^) and Si–O–Si (466 cm^−1^) bonds [49,50,51,52,53].

The spectrum of neomycin sulfate is characterized by a broad band of 2400 3600 cm^−1^, which corresponds to numerous bond vibrations typical of organic compounds: stretching vibrations of N–H bonds in amino groups in the range of 3100–3600 cm^−1^ and stretching vibrations of C–H bonds in alkyl groups in the range of 2400–2800 cm^−1^. Distinct bands in the range of 1400–1700 cm^−1^ result from bending vibrations of N–H bonds and stretching C–H and C–N bonds; in this area, stretching vibrations in aromatic rings are also visible (1430–1500 cm^−1^). The band of strong absorption of infrared radiation in the range of 900–1300 cm^−1^ is attributed to the stretching vibrations of C–N and C–O bonds and the deformation of bonds in aromatic rings is manifested in the range of 600–850 cm^−1^ [16,54]. It should be noted that the described characteristic bands of montmorillonite and neomycin sulfate largely overlap, which makes it difficult to assess the degree of drug intercalation on this basis.

In the hydrated reference samples (MMT + H_2_O), no major changes were noted compared to the initial (dry) montmorillonite and slight differences in the intensity of the bands related to the presence of water were noticed only for samples subjected to the hydration process at elevated temperatures (50 and 80 °C).

The comparison of sections of the spectra of the starting raw materials and individual conjugates for characteristic frequency ranges undoubtedly confirms that neomycin was intercalated into the interlayer spaces of montmorillonite. Thus, the wide band of neomycin sulfate in the range of 2400–3200 cm^−1^ resulted in an increase in the absorption intensity of infrared radiation of the conjugates compared to unmodified and hydrated montmorillonite. Moreover, although the 1635 cm^−1^ peak from neomycin coincides with the band for the same frequency in the spectrum of montmorillonite, its intensity increases significantly in conjugates with a higher drug content (1.0 CEC and 1.25 CEC). Additionally, at around 1530 cm^−1^, a band appears in the spectra of the conjugates, absent in the spectrum of montmorillonite, clearly, therefore, originating from neomycin. Moreover, there is a clear change in the shape of the band in the range of 900–1200 cm^−1^ because of the introduction of neomycin. In consequence, the relatively sharp band characteristic of montmorillonite, in the conjugates, broadens as the amount of the drug increases, and its shape increasingly resembles the band present in the spectrum of neomycin. The 620 cm^−1^ peak for neomycin is also visible in the spectra of conjugates, especially those with higher drug content, which is not obvious in the 0.5 CEC conjugates due to the presence of a small band in the montmorillonite spectrum in the same position.

The influence of organic groups derived from the drug on the interaction of conjugates with infrared radiation is greater the greater the initial amount of the drug. This may suggest that a higher drug concentration in the solution ensures the intercalation of a larger amount of drug, even after exceeding the theoretical amount resulting from the ion exchange capacity of montmorillonite. However, there was no significant impact of the intercalation process conditions on the amount of intercalated drug. Regardless of the preparation conditions (mixing time and temperature), the intensities of the bands in the spectra of the conjugates are similar, although it may seem that a slight increase in the intensity of the characteristic bands occurred in the case of conjugates obtained at 50 °C.

### 3.3. Thermal Analysis

DSC/TG analysis allowed for the assessment of the properties of montmorillonite–drug conjugates due to the different behavior of the drug and its carrier with increasing temperature. Montmorillonite is characterized by high thermal stability, while neomycin, as an organic substance, decomposes relatively quickly with increasing temperature. Therefore, the successful intercalation of the drug into the interlayer space of montmorillonite is evidenced by the occurrence of drug-specific effects in the DSC/TG curves for the MMT–NS conjugates.

**Figure 4:**
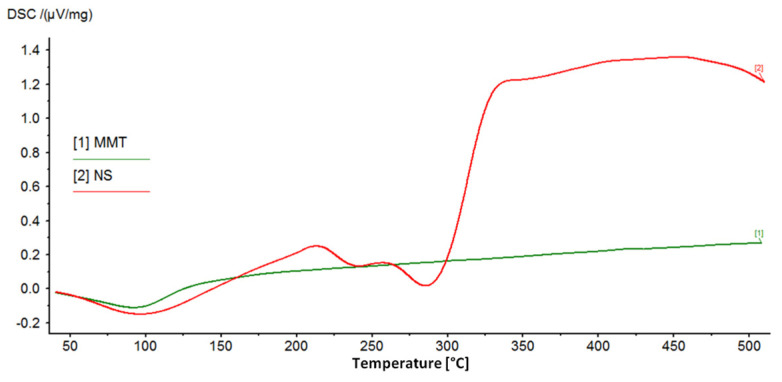
DSC curves of the starting raw materials used to prepare MMT–NS conjugates.

Figure 4 shows the DSC curves recorded for the starting materials used to prepare the conjugates, which clearly show the peaks characterizing the behavior of MMT and NS with increasing temperature. The broad endothermic peak at around 100 °C in the MMT curve corresponds to the loss of water adsorbed on the montmorillonite surface. However, in the measured temperature range, dehydroxylation of the mineral is not visible and the loss of structural water in the form of OH groups is associated with drastic changes in the structure of the mineral and is usually observed at temperatures of 600–750 °C [12,55].

The DSC curve of neomycin sulfate shows two endothermic peaks related to the decomposition (melting) of the drug, for temperatures of 240 °C and 286 °C, and according to literature data, the melting point of neomycin sulfate is 218–237 °C [56]. The final products of the decomposition of neomycin sulfate are primarily nitrogen oxides, CO, CO_2_, and SO_2_ [56]. Next, the organic substance is burned, which is noticeable as a clear exothermic effect.

**Figure 5:**
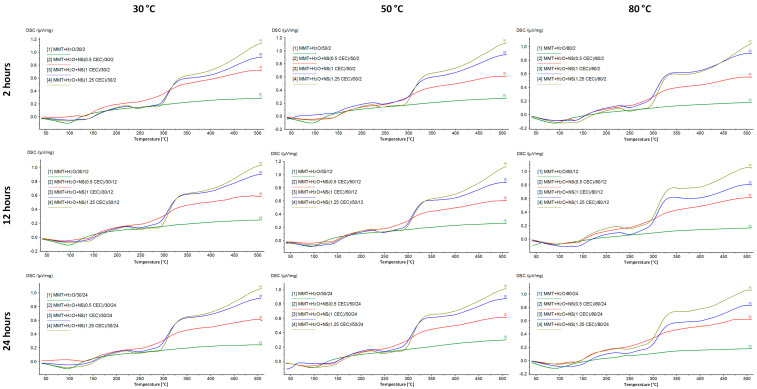
DSC curves of MMT–NS conjugates and the MMT + H_2_O reference sample were obtained under various conditions.

Figure 5 shows DSC curves of MMT–NS conjugates and MMT + H_2_O reference samples. The hydration of montmorillonite has no effect on the curve but the higher the drug content in the conjugate, the more the shape of the curve resembles the curve recorded for neomycin sulfate. In the case of conjugates with a drug content corresponding to 0.5 CEC, the effects of decomposition and combustion of the organic substance are barely noticeable, while for conjugates of 1.0 CEC and 1.25 CEC, they are quite clearly visible.

Moreover, based on the results for the 1.0 CEC and 1.25 CEC conjugates, there was a shift of the peaks corresponding to the drug decomposition toward higher temperatures. The first of them appears at a temperature of 242–244 °C, which, compared to the value recorded for pure neomycin sulfate (240 °C), suggests that the interaction with montmorillonite increased the thermal resistance of the drug. The intercalation process resulted not only in surface adsorption of the drug but also in its binding in the montmorillonite structure, which was also confirmed by the results of X-ray diffractometry and infrared spectroscopy. Increased thermal stability of the organic substance as a result of interaction with the clay carrier has already been reported, e.g., by the authors of studies on the intercalation of nicotine into the interlayer spaces of montmorillonite [20].

The energetic effects associated with the melting and combustion of the drug in the conjugates from the series obtained at 80 °C are much more pronounced than in the case of conjugates obtained at lower temperatures, which may indicate that the thermal decomposition of neomycin most likely began during the intercalation process carried out at 80 °C. Moreover, by comparing the curves of conjugates obtained for 12 and 24 h with those for which the intercalation process lasted 2 h, the longer the intercalation process took place, the more advanced the drug decomposition that occurred. This confirms the previously described results of macroscopic observations, indicating that conjugates prepared at 80 °C were characterized by a clearly darker color, which may indicate the occurrence of thermal transformations of the drug.

Thermogravimetric analysis was performed in parallel with the DSC analysis but, due to the similarity of the obtained curves and the fact that the main information provided by the TG analysis is the sample mass loss, all results are summarized in the form of numerical data in Table 1. For reference, the mass loss of the dry montmorillonite raw material and neomycin were 7.41% and 79.09%, respectively.

In the case of montmorillonite itself, a mass loss of 7.4% was recorded, resulting from the evaporation of water adsorbed by the mineral at a temperature of approximately 100 °C. In turn, neomycin sulfate, as an organic substance, is not resistant to elevated temperatures; hence, above the melting point of the drug (217–238 °C) the slope of the thermogravimetric curve increases rapidly and intensive combustion of the compound begins. At a temperature of 510 °C, the mass loss of the drug reached 79% and the rest were carbonized organic residues.

Reference samples (MMT + H_2_O), regardless of the temperature and duration of the hydration process, showed a smaller mass loss at 510 °C than the starting raw material. In the context of the X-ray diffractometry results (Figure 2), this may mean that during the hydration process, water was bound between the montmorillonite packets, not only adsorbed on the surface and therefore its removal would only be possible at a temperature exceeding 500 °C.

TG measurements performed for MMT–NS conjugates show an obvious dependence of the increase in mass loss with the increase in the initial amount of drug, so the more drug there was in the solution, the more it was incorporated between the montmorillonite packets, even after exceeding the amount of drug corresponding to the ion exchange capacity of the mineral. As the amount of drug increases, the thermogravimetric curves of the conjugates take on a shape that increasingly resembles the shape of the curve recorded for pure neomycin sulfate.

Moreover, it should be noted that the amounts of drug corresponding to the values of 0.5, 1.0, and 1.25 CEC represented approximately 26%, 41%, and 47% of the total weight of the powders from which the conjugates were prepared, respectively. Comparing the measured mass losses of the conjugates with these values, it can be concluded that approximately 70–80% of the neomycin present in the solution was bound to the carrier during the intercalation process. This proves the high efficiency of the intercalation process and, according to the research results quoted in the literature, is a very good achievement [18,24,57].

The relatively smallest mass losses of conjugates were recorded for materials obtained at 30 °C and the results recorded for series obtained at 50 °C and 80 °C were comparable. However, while it can be concluded that increased temperature favors the intercalation of neomycin, the differences in mass losses between samples obtained at different times (within individual temperature series) do not present any trends and the dispersion of these values seems to be random and does not provide any basis to draw conclusions about the optimal duration of the intercalation process.

### 3.4. Zeta Potential

The negative charge of montmorillonite TOT layers in the context of intercalation of organic substances is related to the formation of repulsive electrostatic interactions in the case of anionic substances and attractive electrostatic interactions in the case of cationic substances, which is why it is much easier to incorporate cationic drugs into the interlayer spaces of montmorillonite. Since the Zeta potential provides information about the surface potential of the tested particles, its measurement enables the assessment of the effect of drug intercalation on the charge of montmorillonite-based carriers.

Due to the presence of amino groups, neomycin has a positive Zeta potential of 26.9 mV, while the measured potential of unmodified montmorillonite is −26.9 mV. This result proves that the negative charge on the flat surfaces (001) outweighs the positive charge on the edges of the montmorillonite plates and is in agreement with the literature data [58,59].

**Figure 6:**
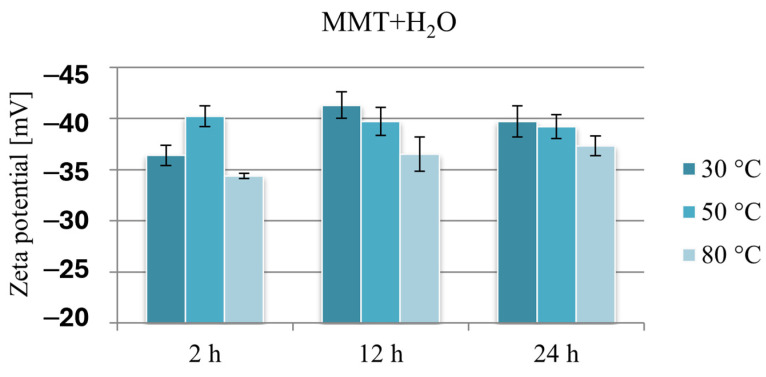
Results of Zeta potential measurements of hydrated reference samples MMT + H_2_O.

Figure 6 compares the Zeta potential values of montmorillonite subjected to hydration under various conditions and clearly shows that because of the hydration process, the negative potential of montmorillonite increases by approximately 10 mV. The lowest potential in absolute value, ranging from −34.4 to −37.3 mV, was recorded in the case of reference samples obtained at 80 °C. The Zeta potential of montmorillonite hydrated at 30 or 50 °C, except for the sample hydrated for 2 h at 30 °C, was in a narrow range from −39.2 to −41.3 mV.

These changes are probably the result of the formation of a “house of cards” structure because of the interaction of montmorillonite layers in aqueous suspensions, and it seems that stirring MMT in an aqueous suspension for 2 h at a temperature slightly above room temperature is an insufficient stimulus for effective hydration of the mineral. Since the hydration of montmorillonite facilitates the intercalation of organic substances into the interlayer spaces, this result may constitute grounds for stating that intercalation will be less effective under these conditions. Similarly, the measurement results of samples obtained at a temperature of 80 °C, at which MMT reached the lowest potential in absolute value, in the context of electrostatic interaction with a cationic drug, may limit the amount of intercalated drug.

**Figure 7:**
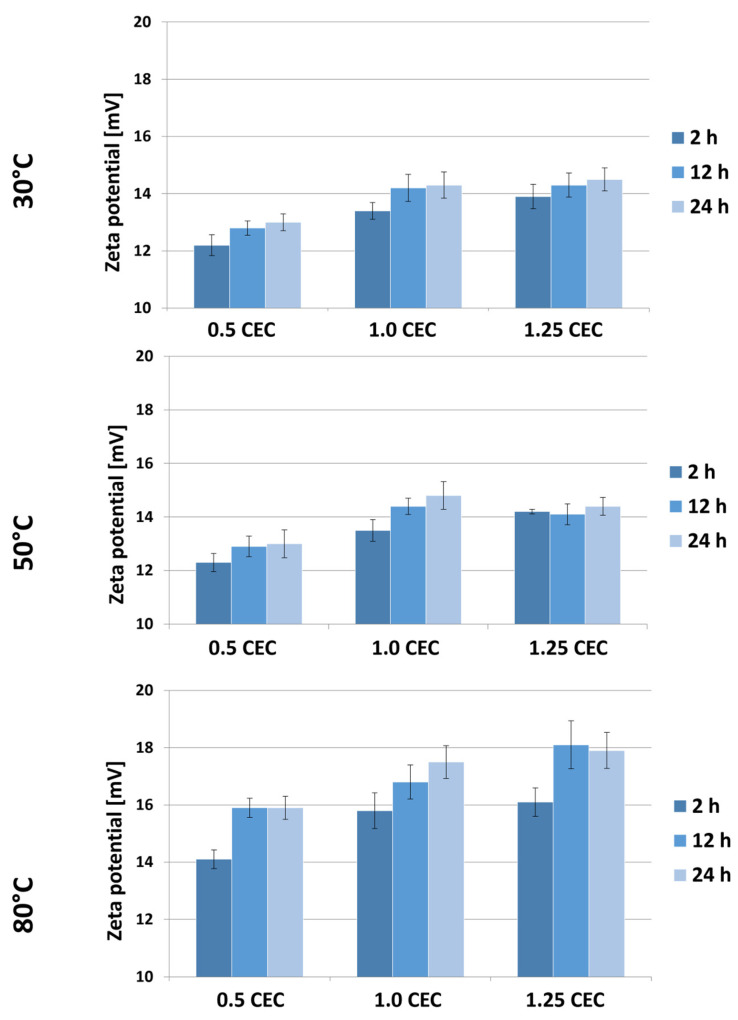
Results of Zeta potential measurements of MMT–NS conjugates obtained under various conditions.

Figure 7 shows the results of Zeta potential measurements for montmorillonite–neomycin conjugates, grouped according to the temperature of the intercalation process. As expected, the presence of neomycin caused a change in the sign of the potential from negative to positive, and a similar effect of the drug on the potential of clay mineral-based carriers was described in a publication on the intercalation of donepezil between smectite mineral packages [18].

Based on the results obtained, a general tendency of increasing Zeta potential with an increase in the initial amount of drug in the solution can be noticed. Moreover, the Zeta potential of the conjugates is greater the higher the temperature and the duration of the intercalation process. Nevertheless, the measured potential values of materials obtained at 30 and 50 °C are similar and range from 12.2 to 14.8 mV. Significantly higher values, ranging from 14.1 to even 18.1 mV, were recorded for a series of conjugates obtained at 80 °C. However, in the face of the observed changes in the color of these materials, it can be assumed that such a significant change in potential was caused by the decomposition products of neomycin or that no effective intercalation occurred at this temperature, so some of the drug was bound on the surface and it being released had a stronger impact on the growth of carrier potential.

As CEC increases, water penetration of the interlaminar spaces becomes easier and, due to temperature, there is faster ion exchange and the possibility of intercalation with neomycin sulfate. Increasing the heating time at a high CEC (1.25) has the effect of stabilizing (saturating) MMT with neomycin sulfate; this can be associated with three phenomena: (a) effective intercalation (the intercalated compound does not affect changes in zeta potential while in the inter-packet space), (b) permanent chemisorption of neomycin to the montmorillonite surface (may promote dynamic equilibrium between adsorbed and desorbed ions on the surface), and (c) heating may stabilize the pH of the suspension, which has a direct effect on zeta potential. A stable pH leads to stabilization of the surface charge of particles (which may be true looking at the pH value of conjugate suspensions). In addition, neomycin sulfate itself is most stable at 56 °C, which may indicate that the stability of the neomycin sulfate water system is also important here (higher heating does not cause changes in either intercalation or surface adsorption capacity).

### 3.5. Drug Release In Vitro

The key issue from the point of view of the use of montmorillonite in neomycin-controlled release systems is the assessment of the release profile of antibiotics from the prepared carriers, which was performed spectrophotometrically using the oxidation reaction of aminoglycoside antibiotics with KMnO_4_.

**Figure 8:**
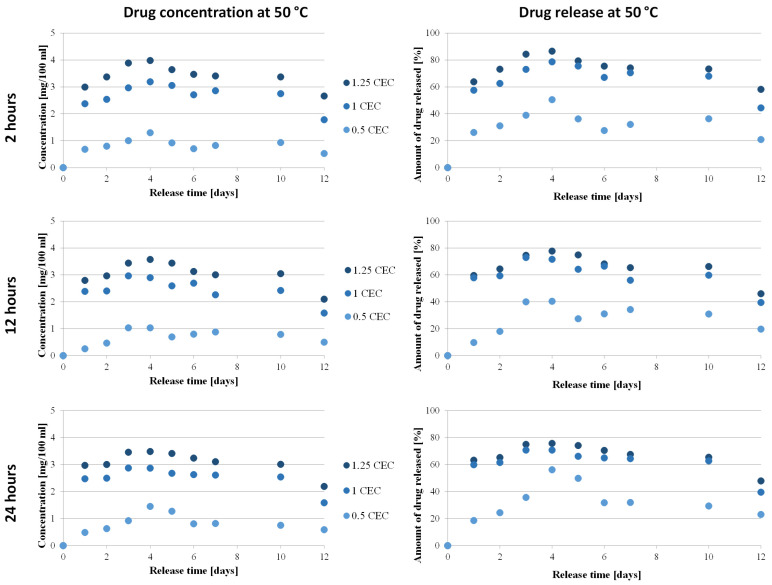
Drug release profile from MMT–NS conjugates obtained under various conditions (drug concentration–time dependence and dependency of the amount of drug released on time).

The relationship between the concentration of neomycin and the amount of drug on its release time from the carrier was determined only for conjugates obtained at 50 °C for 2, 12, and 24 h, during which conditions were considered optimal. The concentrations were determined based on the calibration curve and then converted into the amount of drug released, and the results obtained are presented in Figure 8.

The antibiotic release profiles plotted for the 1.0 CEC and 1.25 CEC conjugates are very similar to each other and are characterized by the occurrence of an initial phase of rapid release of the drug (so-called burst) that was only bound on the montmorillonite surface, during which approximately 60% of the antibiotic was released. This is not a beneficial or desirable phenomenon in controlled drug delivery systems but it is often described in publications on the use of clay minerals as drug carriers [13,18,22,60]. Apparently, the reactivity of the montmorillonite surface causes some of the organic substances to be intercalated to be bound on the surface before the compound is incorporated between the mineral packets. From the second day, neomycin is gradually and stably released from the carriers and the plateau phase with slight fluctuations lasts until the 10th day, with the concentration of neomycin during release from the 1.0 CEC and 1.25 CEC carriers remaining at the level of 2.4–3.2 mg/100 mL and 2.8–4.0 mg/100 mL, respectively. The amount of drug released from the 1.0 CEC and 1.25 CEC conjugates reaches approximately 70–80%, with the highest amount recorded for conjugates obtained at 50 °C for 2 h (maximum 86%). Since the ion exchange reaction is an equilibrium process, there is no complete exchange of ions located in the interlayer spaces; therefore, the release of the drug from the clay carrier was never 100% [15].

In the case of 0.5 CEC conjugates, the plotted relationship between the amount of drug released and the release time is less stable but it is visible that during the first day, 26%, 10%, and 19% of neomycin was released from the carriers obtained for 2, 12, and 24 h, respectively. Unfortunately, in the further release phase, the amount of released drug reaches a maximum of 56%, which is a much worse result than that obtained in the case of the 1.0 CEC and 1.25 CEC conjugates. Additionally, significant fluctuations in drug concentration were noted (in the range from 0.3 to 1.5 mg/100 mL), especially in the case of conjugates obtained at 50 °C for 24 h. A clear maximum was recorded on the fourth day after the start of the experiment, and until the 10th day, the amount of drug released remained at approximately 30%. Therefore, much more favorable profiles from the point of view of sustained antibiotic release were recorded for the 1.0 CEC and 1.25 CEC conjugates.

The minimum inhibitory concentration (MIC) of neomycin was determined using broth microdilution according to the recommendations of the Clinical Laboratory Standards Institute (CLSI, 2017), using *Escherichia coli* ATCC 25922 as the quality control in tests performed in triplicate, provided the critical points defined by the Comite de L’Antibiogramme de la Société Française de Microbiologie are S ≤ 0.8 mg/100 mL and R >1.6 mg/100 mL [61]. The results of in vitro drug release measurements showed the potential to maintain the drug concentration at 1.0–4.0 mg/100 mL for a prolonged period of time, widening the therapeutic window, thus confirming the ability of montmorillonite to modify the release profile of neomycin toward a sustained release.

### 3.6. Antibacterial Activity

**Figure 9:**
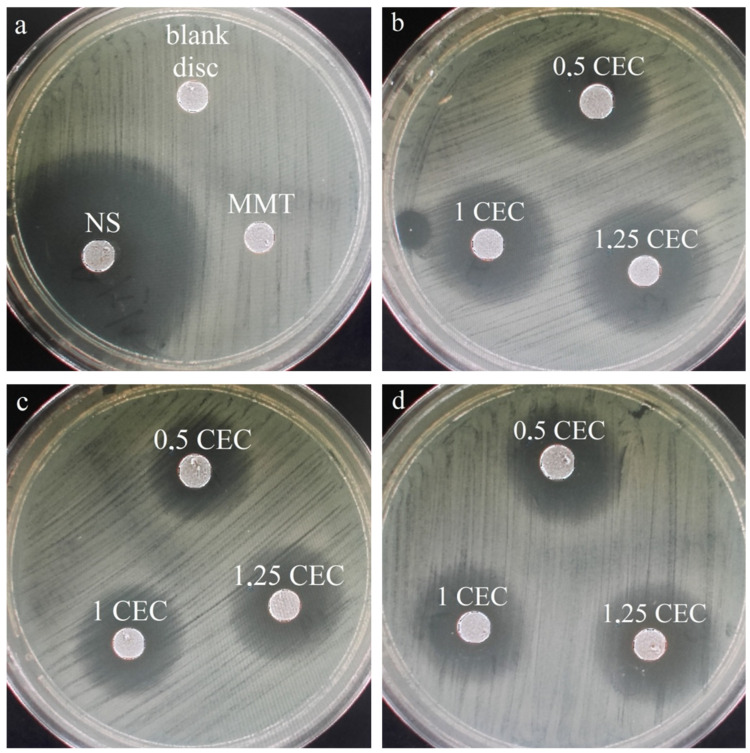
Zones of inhibition of the growth of *E. coli* bacteria for (**a**) montmorillonite (MMT), neomycin sulfate (NS), and a disc containing no antibiotic (blank disc) and (**b**) a series of MMT–NS conjugates obtained at a temperature of 50 °C for 2 h (**b**), 12 h (**c**), and 24 h (**d**).

Similarly to drug release studies, antibacterial tests were performed for a series of conjugates obtained at 50 °C only, which seemed to be the most promising based on the results of physicochemical tests of the carriers. For comparison and objectivity, the test was also conducted for the starting raw materials, i.e., MMT and NS and the measured diameter of the *E. coli* growth inhibition zone for neomycin sulfate was 38 mm, while no inhibition zone was observed around the montmorillonite disc. The latter result is not surprising and the lack of antibacterial activity of unmodified montmorillonite has been previously described in the literature [22,23,24]. Photos of the bacterial growth inhibition zones for all tested materials are shown in Figure 9 and the results of measurements of the diameter of the bacterial growth inhibition zone around the MMT–NS discs are presented in Table 2.

The occurrence of zones of inhibition of bacterial growth around the conjugate discs indicates that the antibiotic is effectively desorbed from montmorillonite and diffuses in the agar and that the antibacterial activity of the drug has not been adversely affected in any way because of the interaction with the clay carrier. Similar results have been described for montmorillonite intercalated with antibiotics such as chlorhexidine [22,24] and tetracycline and minocycline [23].

The largest zones of bacterial growth inhibition were observed for samples prepared for 2 h and especially for the conjugate with the initial amount of drug in solution corresponding to 1.25 CEC of montmorillonite. For this specific material, the zone of inhibition of bacterial growth was 19 mm, so it reached half the diameter of the inhibition zone around the disc of paper soaked in neomycin sulfate solution (blank sample). In the case of materials obtained for 2 and 12 h, an increasing tendency in the diameter of the inhibition zone with an increase in the initial amount of the drug is visible, which confirms previous observations that the more drug is in the solution in which montmorillonite is stirred for intercalation, the more drug is incorporated into the interlayer spaces, even after exceeding the theoretical amount resulting from the ion exchange capacity of the mineral.

Although the same tendency is noticeable in the case of conjugates obtained for 12 h, the diameters of the bacterial growth inhibition zones are on average 5–10% smaller than those around the disc from conjugates obtained for 2 h. The relatively weak antibacterial effect was demonstrated by materials obtained within 24 h, which may indicate that such a long duration of the intercalation process at elevated temperatures causes a slow decomposition of neomycin, and therefore, less of the drug retains its antibacterial properties.

## 4. Conclusions

The paper presents research results on the possibility of using montmorillonite as a neomycin carrier and confirms that montmorillonite–neomycin conjugates (MMT–NS), obtained for 2 to 24 h in an aqueous solution at 30, 50, and 80 °C with the initial amount of dissolved drug in the solution corresponding to the values of 0.5, 1.0, and 1.25 CEC of montmorillonite, can be successfully used in the sustained drug release.

The effectiveness of neomycin intercalation in montmorillonite was confirmed by X-ray diffractometry, FTIR spectroscopy, and thermal analysis and it was found that neomycin was incorporated between the packages in the form of a monolayer and not only on the surface of the TOT montmorillonite layer, which was also confirmed by the results of thermal analysis. Zeta potential measurements also showed that the presence of the drug causes the charge of the carriers to change from negative, typical of montmorillonite, to positive.

The efficiency of the intercalation process was estimated at 70–80%. The amount of neomycin injected into MMT can be higher or even exceed the theoretical value resulting from the ion exchange capacity of MMT. This is due to the possibility of the formation of MMT–drug conjugates on the edges and outer surfaces.

The influence of temperature on the amount of intercalated drug is clear because the hydration of montmorillonite facilitates the penetration of interlayer spaces by foreign molecules and the thermal energy accelerates the hydration of MMT, which, with an increase in temperature, creates more favorable conditions for intercalation. However, too high a temperature may lead to progressive decomposition of the drug already during the intercalation process, which was confirmed by macroscopic observations of the obtained conjugates and DSC analysis, and was therefore considered the optimal intercalation temperature.

Therefore, only MMT–NS conjugates obtained at a temperature of 50 °C were tested for in vitro drug release and antibacterial activity, which confirmed the ability of montmorillonite to modify the release profile of neomycin toward prolonged release, while showing high antibacterial activity, which was confirmed by the presence of zones of inhibition of the growth of Gram-negative *Escherichia coli* bacteria around the MMT–NS conjugate discs. The latter studies also showed that the antibiotic was effectively desorbed from montmorillonite and the interaction with the clay carrier did not affect the bactericidal effect of the drug.

It is worth noting that the duration of the intercalation process did not significantly affect the quantity but it did affect the quality of the intercalated drug; based on the results of antibacterial tests, it was found that conducting the intercalation process for 24 h at a temperature of 50 °C adversely affects the bactericidal properties of neomycin. It is rather clear that 2 h at moderate temperature is sufficient for effective intercalation of the drug into the interlayer spaces of montmorillonite; the materials prepared under these conditions (50 °C, 2 h) showed the largest zones of inhibition of the growth of *E. coli* bacteria and a favorable drug release profile and also the largest amount of drug released.

To sum up, it can be stated that the drug release and antibacterial response studies performed on the obtained conjugates confirmed the potential of montmorillonite for use in controlled neomycin release systems.

## Figures and Tables

**Table 1:** Percentage mass loss of MMT–NS conjugates at 510 °C.

Temperature [°C]	Mixing Time [h]	Mass Loss [%]			
		MMT + H_2_O	0.5 CEC	1.0 CEC	1.25 CEC
30	2	6.98	18.13	33.45	38.26
	12	6.94	18.94	33.32	38.16
	24	7.08	16.10	32.93	38.33
50	2	7.28	18.64	34.25	37.93
	12	7.34	19.15	33.34	38.62
	24	7.29	19.46	34.23	38.26
80	2	6.44	19.48	34.00	38.81
	12	6.77	18.75	34.44	38.59
	24	6.79	19.09	32.78	38.44

**Table 2:** Results of measurements of the diameter of the zones of inhibition of *E. coli* bacteria growth for a series of conjugates obtained at a temperature of 50 °C (average value from three samples).

Temperature	Mixing Time [h]	Zone of Inhibition [mm]		
		0.5 CEC	1.0 CEC	1.25 CEC
50 °C	2	16	18	19
	12	14	16	18
	24	17	15	17

## Data Availability

All data are presented in the current manuscript. For further queries, communicate with the corresponding authors.
